# Supplementary material for: Osteogenic-Inducing Apatite/Agarose/Gelatin Hybrid Scaffolds Embedding Gold Nanoparticles
Source: Pharmaceutics. 2025 Aug 25;17(9):1103. doi: 10.3390/pharmaceutics17091103 (PMC12472558; doi:10.3390/pharmaceutics17091103)
Supplement: Supplementary file 1 [file pharmaceutics-17-01103-s001.zip › pharmaceutics-3801344-supplementary.pdf]

Supporting Information

Osteogenic-Inducing Apatite/Agarose/Gelatin Hybrid Scaffolds Embedding Gold Nanoparticles

María Victoria Cabañas [1,2], Paola S. Padilla [1], Mónica Cicuéndez [1,3,4], Sandra Sánchez-Salcedo [1,2,4], Jesús Román [1,2,*] and Juan Peña [1,2,*]

1 Departamento de Química en Ciencias Farmacéuticas, Facultad de Farmacia, Universidad Complutense de Madrid, UCM, 28040 Madrid, Spain; vcabanas@ucm.es (M.V.C.); paola-padillava@uide.edu.ec (P.S.P.); mcicuend@ucm.es (M.C.); sansanch@ucm.es (S.S.-S.)

2 Instituto de Investigación Hospital 12 de Octubre, i+12, 28040 Madrid, Spain

3 Instituto de Investigación Sanitaria del Hospital Clínico San Carlos (IdISSC), 28040 Madrid, Spain

4 CIBER de Bioingeniería, Biomateriales y Nanomedicina (CIBER-BBN), 28040 Madrid, Spain

* Correspondence: jeromzar@ucm.es (J.R.); juanpena@ucm.es (J.P.)

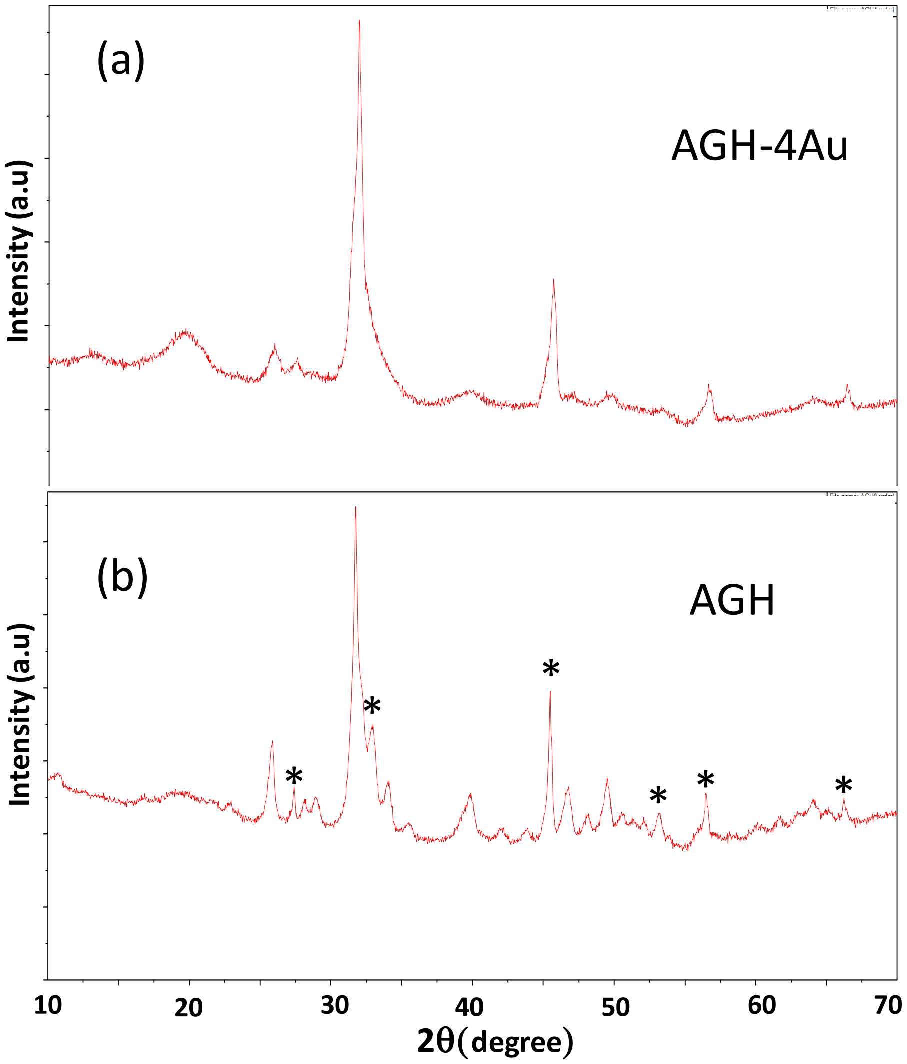

Figure S1. XRD patterns corresponding to scaffolds fabricated in PBS. (a) AGH-4Au scaffold prepared with GNPs-PEG; (b) AGH scaffold prepared without NPs (* indicate diffraction maxima corresponding to NaCl)

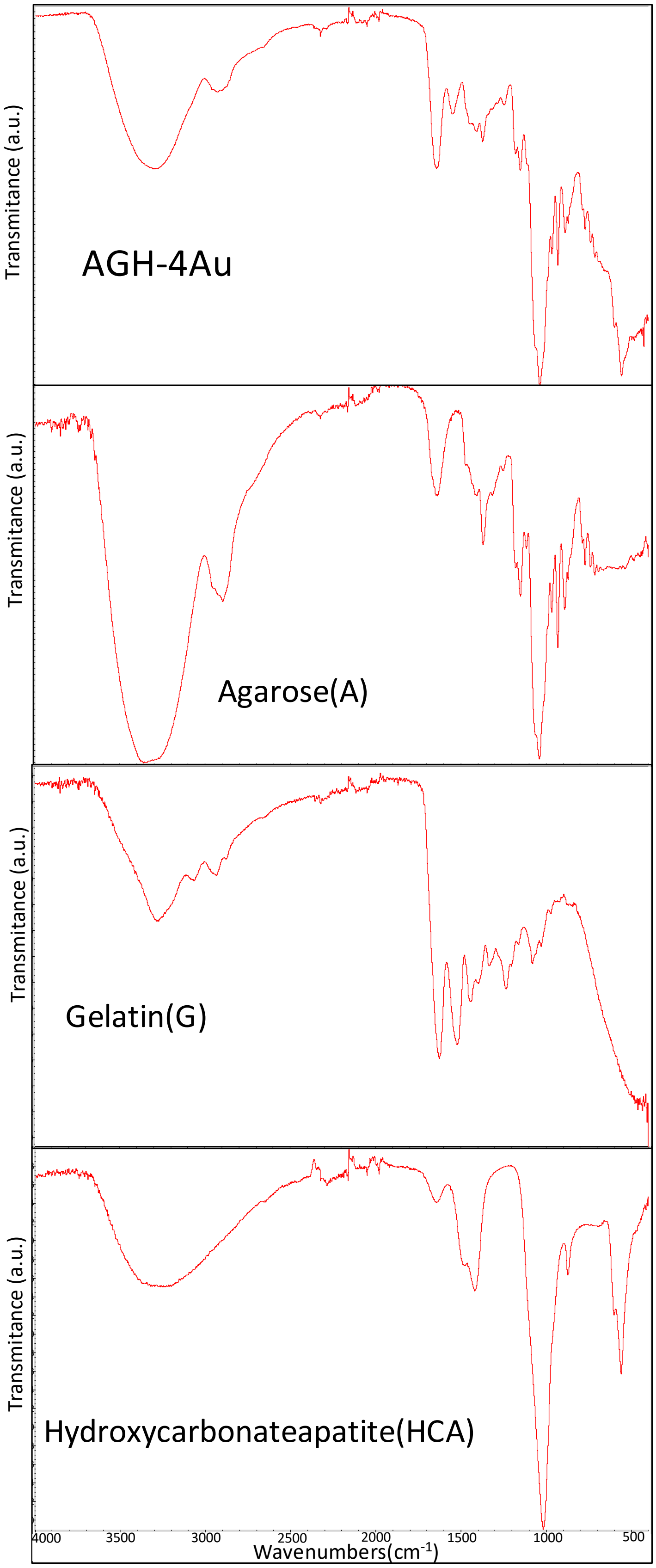

Figure S2. FTIR spectra corresponding to AGH-4Au scaffold fabricated in PBS as well as their components: Agarose, gelatin and hydroxycarbonateapatite
